# Go-stimuli proportion influences response strategy in a sustained attention to response task

**DOI:** 10.1007/s00221-016-4701-x

**Published:** 2016-06-21

**Authors:** Kyle M. Wilson, Kristin M. Finkbeiner, Neil R. de Joux, Paul N. Russell, William S. Helton

**Affiliations:** 1 Department of Psychology, University of Canterbury, Private Bag 4800, Christchurch, New Zealand; 2 Human Factors Research Group, Faculty of Engineering, University of Nottingham, Nottingham, UK; 3 The Applied Cognition and Cognitive Engineering Group, Department of Behavioural and Social Sciences, University of Huddersfield, Huddersfield, UK

**Keywords:** Sustained attention, Response inhibition, SART, Speed–accuracy trade-off, Task-related thought, Task-unrelated thought

## Abstract

The sustained attention to response task (SART) usefulness as a measure of sustained attention has been questioned. The SART may instead be a better measure of other psychological processes and could prove useful in understanding some real-world behaviours. Thirty participants completed four Go/No-Go response tasks much like the SART, with Go-stimuli proportions of .50, .65, .80 and .95. As Go-stimuli proportion increased, reaction times decreased while both commission errors and self-reported task-related thoughts increased. Performance measures were associated with task-related thoughts but not task-unrelated thoughts. Instead of faster reaction times and increased commission errors being due to absentmindedness or perceptual decoupling from the task, the results suggested participants made use of two competing response strategies, in line with a response strategy or response inhibition perspective of SART performance. Interestingly, performance measures changed in a nonlinear manner, despite the linear Go proportion increase. A threshold may exist where the prepotent motor response becomes more pronounced, leading to the disproportionate increase in response speed and commission errors. This research has implications for researchers looking to employ the SART and for more applied contexts where the consequences of response inhibition failures can be serious.

## Introduction

The sustained attention to response task (SART; Robertson et al. 1997) is a high Go, low No-Go response task developed to measure sustained attention in patients with traumatic brain injury to the frontal lobes. Many studies have used the SART as a measure of sustained attention (Chan 2001, 2002; Greene et al. 2009; Smallwood et al. 2003, 2007). Typically, simple number stimuli (e.g. 1–9) have been used in the SART. Participants are tasked with responding to Go stimuli occurring 89 % of the time (numbers 1–9, except for 3), and to withhold responses to rarely occurring No-Go stimuli (the number 3). Performance is measured primarily by errors of commission (inappropriately responding to a No-Go stimulus), errors of omission (inappropriately failing to respond to a Go stimulus) and reaction time to Go stimuli. Errors of commission normally occur much more frequently (30–50 %) than errors of omission (5–10 %) in the SART (Carter et al. 2013; Head and Helton 2012). Further, performance is typically characterized by a speed–accuracy trade-off: people who respond faster to Go stimuli also inappropriately respond more often to No-Go stimuli (Helton 2009; Helton et al. 2005).

There is ongoing debate regarding the mechanism responsible for errors of commission in the SART. One perspective is that commission errors occur because of the monotonous nature of SART stimuli and the task itself. The task induces feelings of boredom and mind-wandering (Smallwood and Schooler 2006) or a state of mindlessness (Manly et al. 1999; Robertson et al. 1997) which in turn results in perceptual decoupling (failures to recognize the No-Go stimuli) and an automatic pattern of responding that requires little effort but is responsible for more commission errors. From the mind-wandering perspective, this is said to be evidenced by an increase in task-unrelated thoughts. Proponents of the perceptual decoupling interpretation do recognize that the speed–accuracy trade-off is a major feature of the SART; however, they attribute this to the supposed decoupling of conscious perception from the task, which induces faster responding to the Go stimuli and the inability to withhold responses to the No-Go stimuli (Manly et al. 1999; Robertson et al. 1997; Smallwood et al. 2004).

A competing explanation is that the trade-off between speed of response to Go stimuli and the risk of responding to No-Go stimuli is the result of a deliberate response strategy and not decreased external awareness of the identity of stimuli per se (Peebles and Bothell 2004). While SART instructions typically place equal emphasis on accuracy and response speed, participants may favour a strategy that maximizes speed over accuracy. Indeed, they may switch back and forth between these strategies dynamically (Head and Helton 2014). With 89 % “Go” trials and only 11 % “No-Go” trials, the benefit of speed on 89 % of trials may outweigh the costs to speed of slowing sufficiently on all trials to avoid inappropriate responses to No-Go stimuli (commission error) on only 11 % of trials. Peebles and Bothell (2004) show that an adaptive control of thought-rational (ACT-R; Anderson and Lebiere 1998) model that incorporates two competing response strategies is able to successfully predict observed relationships between SART reaction times to Go stimuli and probability of commission errors. The two strategies in their model of SART performance are labelled “encode and click” (respond) and “encode and check”. In the encode and click strategy, the participant does not wait to analyse the contents of the stimuli but simply responds to the presence of any stimulus as quickly as possible. This strategy maximizes speed (participants are instructed to respond quickly), which 89 % of the time is an effective strategy in the SART. Conversely, the encode and check strategy slows the response to all stimuli because it requires subjects to verify the identity (or at least response category) on all trials. This strategy results in slower responses on all trials, but facilitates the appropriate withholding of a response to No-Go stimuli. The strategy choice is dynamic, in that subjects may switch between the two. Rather than adopting one strategy though, they revise the strength of each strategy after each success or failure and make adjustments accordingly. For example, after a commission error the utility of “check” is enhanced, and after a fast correct Go response, “click’s” utility is boosted. This supports the idea that subjects are perceptually aware of the task, as opposed to decoupled during the task.

The strategy choice is likely influenced by multiple factors, such as top-down control and individual differences. Prior research has found, for example, that simply altering the task instructions to emphasize either speed or accuracy has a marked effect on task performance, indicating the role of top-down control or strategy choice (Seli et al. 2012). In addition to top-down strategy choice, task characteristics will affect the strategy adopted. For example, Head and Helton (2013, 2014) were able to artificially slow responses down (and thus reduce commission errors) by requiring participants to first move a mouse pointer towards stimuli before they had the opportunity to click to respond.

Another task characteristic likely to influence strategy choice would be the relative proportion of Go stimuli to No-Go stimuli. The encode and click strategy (emphasizing response speed) should be biased to occur when there are higher Go-stimuli proportions, as a high-speed response strategy is maximally beneficial when Go stimuli are more prevalent. This should also result in overall faster response rates in the task with higher relative Go-stimuli proportions. The encode and check strategy (emphasizing accuracy) should, however, be biased to occur when there are relatively more No-Go stimuli, as the alternative strategy emphasizing speed would result in more errors in this setting. This would result in a switch towards the slower encode and check strategy, and this would result in slower response rates to the Go stimuli. Closely examining behaviour while varying Go-stimuli proportion in the SART may shed further light on the debate between the perceptual decoupling and response strategy perspectives.

The SART, while an interesting research puzzle in and of itself given the competing theoretical perspectives, may also prove useful in understanding real-world behaviour. Wilson et al. (2014) conducted a simulated firearms task utilizing a Go/No-Go paradigm. Participants confronted a mixture of foes (Go stimuli) and friends (No-Go stimuli) in a simulation of a military or law enforcement scenario using human actors. They used proportions of high Go (.89), low Go (.11) and medium Go (.50). They found that participants failed to withhold responses (committed errors of commission or friendly fire) more often as the Go (foe) proportion increased. Interestingly, despite the Go proportion increase being linear, the increase in errors of commission was accelerating (not constant linear). There was no difference in commission errors between low Go and medium Go proportions; however, there was a large difference between the medium Go and high Go proportions. They were unable to measure response speed though so it was not clear what relationship this had with Go-stimuli proportions. They went on to suggest that a breaking point or a threshold of sorts may exist, wherein a prepotent motor response (see Head and Helton 2014; Helton 2009; Helton et al. 2010; Robertson et al. 1997) takes precedence after the proportion of Go responses exceeds equal probability (.50). If such a threshold exists, it would be highly useful to be able to use response proportion to predict when a prepotent motor response may take effect and seriously hamper people’s ability to inhibit subsequent responses when required. In the context of friendly fire for instance, a law enforcement or military commander may be able to use knowledge of a combat zone to help predict when friendly fire accidents are at a particularly high risk of occurring.

The current experiment aimed to further explore how relative Go-/No-Go-stimuli proportion affects performance in the SART. To do this, a number of different proportions were used in a computer-based Go/No-Go task. Following suggestions that the Go proportion’s most influential effects on response inhibition occur somewhere upwards of the 50 % Go-stimuli proportion (Wilson et al. 2014), four conditions beginning at 50 % Go stimuli and increasing in equal intervals through to 95 % Go stimuli were used. Participants completed all four tasks in a repeated-measures design. For each condition, the performance metrics of commission errors (failures to withhold to No-Go stimuli), omission errors (failures to respond to Go stimuli) and reaction times to Go stimuli were recorded along with a questionnaire to measure participants’ task-related and task-unrelated thoughts.

As the Go-stimuli proportion increases from .50 to .95, the opportunity to correct the speed-beneficial “click” in favour of “check” will occur less often. Considering the two theories of SART performance—perceptual decoupling and response strategy—both might predict that reaction times to Go stimuli will decrease and errors of commission rates will increase. A proponent of the response strategy perspective would argue this is because of the relative success of the two response strategies in conditions of differing Go-stimuli probability. Conversely, a proponent of the perceptual decoupling perspective may suggest this is because high proportions of Go stimuli lull participants into a more automatic disposition towards the task, which allows increased mind-wandering and mindlessness.

However, while the two perspectives may predict manipulations of Go-stimuli probability to have identical effects on reaction times and rate of commission errors, different predictions are made for self-reported incidences of task-related and task-unrelated thoughts. The two perspectives would differ in regard to the impact of differing Go-stimuli proportions on self-reports of task-related and task-unrelated thoughts. Within the perceptual decoupling perspective, a mindlessness proponent might hypothesize both task-related and task-unrelated thoughts will decrease in tasks with higher Go-stimuli proportions, as the higher Go-stimuli proportions would result in a reduction in overall conscious awareness (mindlessness) due to increased automaticity. Alternatively (but still within the perceptual decoupling perspective), a mind-wandering proponent might suggest task-unrelated thoughts will increase and task-related thoughts will decrease with increasing Go-stimuli proportions. In addition, proponents of a mind-wandering perspective would also suggest a positive correlation between reports of task-unrelated thoughts and commission error rates and a negative relationship between task-unrelated thoughts and response time to Go stimuli. From the response strategy perspective, the person is fully aware of their ongoing performance during the task. This is evidenced by subjects “self-correcting” following errors of commission in the SART; reaction times increase following commission errors (Manly et al. 2000). Participants must be attentive to their commission errors to be able to correct for them, which they appear to do by altering their response strategy. Further, McAvinue et al. (2005) found that participants were aware of their commission errors 99.1 % of the time. A proponent of the response strategy theory would suggest that increased commission errors occurring due to higher Go-stimuli proportions would instead result in increased concern and thoughts regarding task performance. Reports of task-related thoughts should increase in higher Go-stimuli proportion conditions, as failures to appropriately withhold are very salient. Finally, if a threshold exists wherein the prepotent motor programme disproportionately increases in efficacy after Go-stimuli probability surpasses a certain level, any increase in commission errors might be best characterized as an accelerating function as opposed to a constant linear function.

## Methods

### Participants

Participants were 30 (12 males and 18 females) undergraduate students from the University of Canterbury, Christchurch, New Zealand. They ranged in age between 20 and 54 years (*M* = 26.5, *SD* = 7.8). All had normal or corrected to normal vision, and their participation was part of a course requirement.

### Materials and procedure

Participants were tested in individual workstation cubicles, seated 50 cm in front of Phillips 225B2 LCD computer screens (1680 × 1050 pixels, 60 Hz refresh rate) mounted at eye level. All stimulus and response timing were controlled using E-prime 2.0 software (Psychology Software Tools, Pittsburgh, PA) running on 3.40 GHz Intel i7 2600 PC computers. Head movements were not restrained. Any wrist watches were removed, and mobile phones were switched off. Go/No-Go tasks that were modified versions of the SART (Robertson et al. 1997) were used. The original SART uses a Go proportion of .89 (No-Go proportion of .11). We used four variations on this proportion in a repeated-measures design: .50, .65, .80 and .95. Each SART consisted of 208 stimuli presentations. Images of robots (approximately 85 mm × 85 mm) were used as Go and No-Go stimuli. One robot was an XM1219 Armed Robotoc Vehicle (https://en.wikipedia.org/wiki/XM1219_Armed_Robotic_Vehicle#/media/File:FCS-MULE-ARV-2007.jpg), and the other was a Legged Squad Support System (https://it.wikipedia.org/wiki/Legged_Squad_Support_System#/media/File:Leggedsquadsupportsystem0.png). Typically digit stimuli (1–9) have been used in the SART. This works well when solely using the original SART Go proportion of .89, as one of the nine digits (typically 3) is used as a No-Go stimulus and therefore all digits have the same chance of occurring (.11). In the current experiment however—because participants were required to respond to different proportions of stimuli over the four SARTs—having various different digits representing No-Go stimuli would likely have been confusing as participants switched between Go-stimuli proportions. We wanted to avoid any additional cognitive load that may have been caused by participants trying to remember which stimuli were Go stimuli and which were No-Go stimuli on any given SART. As well as the original images, mirror images of both robots were used for half of stimuli presentations to help ensure participants did not simply respond to a basic shape template. Images were used instead of numbers to provide more realism necessary for the future application of the Go/No-Go task to Shoot/Don’t-Shoot tasks (see Wilson et al. 2015b). In addition, a simpler set of stimuli should actually be more likely to induce the perceptual decoupling and mindlessness proposed by some SART researchers (see Head and Helton 2012). Other authors have successfully incorporated nondigit stimuli into the SART, such as Smallwood et al. (2009) who used “O” as a Go stimulus and “=” as a No-Go stimulus (see also Smallwood 2013). Two 16-item subscales of the Dundee Stress State Questionnaire (DSSQ; Matthews et al. 1999, 2002) were administered to collect reported thoughts before and after the tasks. Participants answered using a 5-pt Likert scale anchored with “never” (1) and “very often” (5). One subscale measured levels of task-related thoughts, while the other measured levels of task-unrelated thoughts.

After being seated at their workstations, participants completed a pre-task DSSQ. Pre-task measures were used as baselines for which to later compare with each of the post-task thought measures. To answer the pre-task measures, participants were told to “Please indicate roughly how often you had each thought DURING THE LAST TEN MINUTES”. After completing the pre-task DSSQ, participants were informed that they would be completing four separate Go/No-Go response tasks. Through random assignment, half of participants had the XM1219 Armed Robotoc Vehicle as the Go stimulus and the Legged Squad Support System as the No-Go stimulus, while the other half had the opposite. Participants’ Go- and No-Go-stimuli assignment remained the same for all four SARTs. Stimuli in each Go probability condition were presented in a different random order for each participant. Participants were instructed to respond, by pressing the spacebar, to Go stimuli and to withhold responses to No-Go stimuli. They were told to respond as fast and accurately as possible (speed and accuracy were emphasized equally). A practice task was administered before the first of the four trials began. This consisted of 20 trials with a Go-stimuli proportion of 50 %. Verbal accuracy feedback was given after each trial. The order in which tasks were completed was random. Immediately before each task began, participants were informed of what the proportion of Go stimuli in the following task was to be. Stimuli were presented centrally on the screen for 250 ms, followed by a 900-ms mask consisting of a circle (29 mm in diameter) with a diagonal line through it. Thus, there was an 1150-ms stimuli onset to stimuli onset interval. Responses were recorded up to 900 ms following stimuli onset. Each task lasted approximately 4.3 min. Immediately after each task participants completed a post-task DSSQ. For each of the post-task DSSQs, participants were told “This questionnaire is concerned with your feelings and thoughts DURING THE TASK that you have JUST COMPLETED”. The whole experiment took approximately 28 min.

## Results

Results from 2 of the 30 participants were removed due to both having excessive amounts of errors (both omission and commission), indicating that they had failed to follow task instructions.

### SART performance

**Fig. 1:**
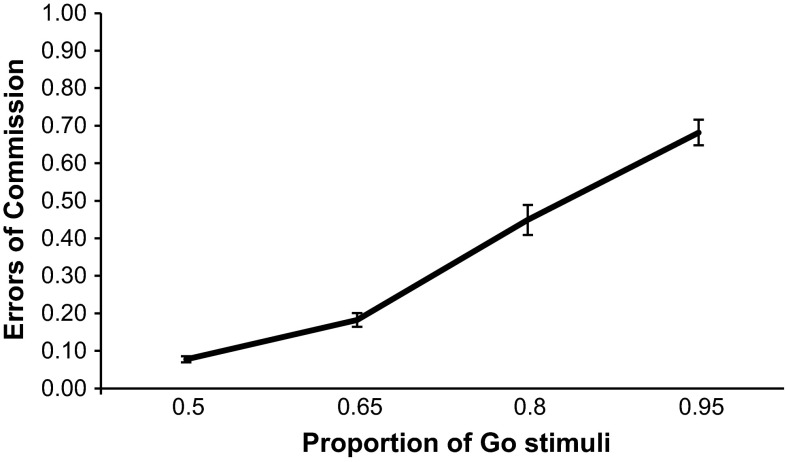
Mean proportion of errors of commission for each Go-stimuli proportion. *Error bars* are standard errors

**Fig. 2:**
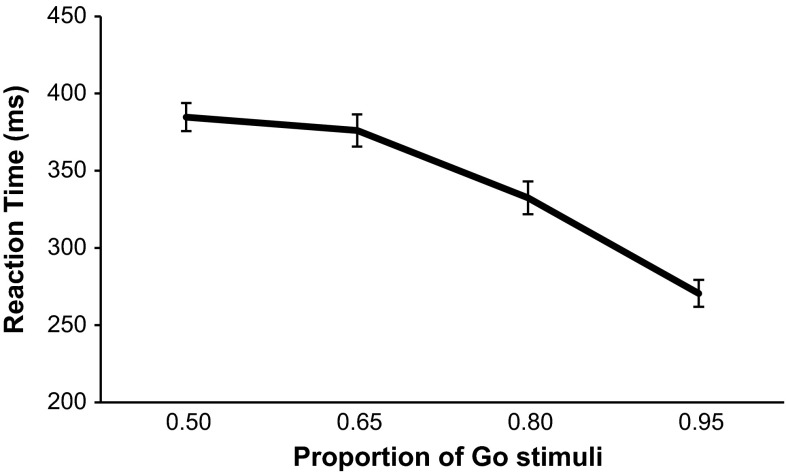
Mean reaction time for each Go-stimuli proportion. *Error bars* are standard errors

**Fig. 3:**
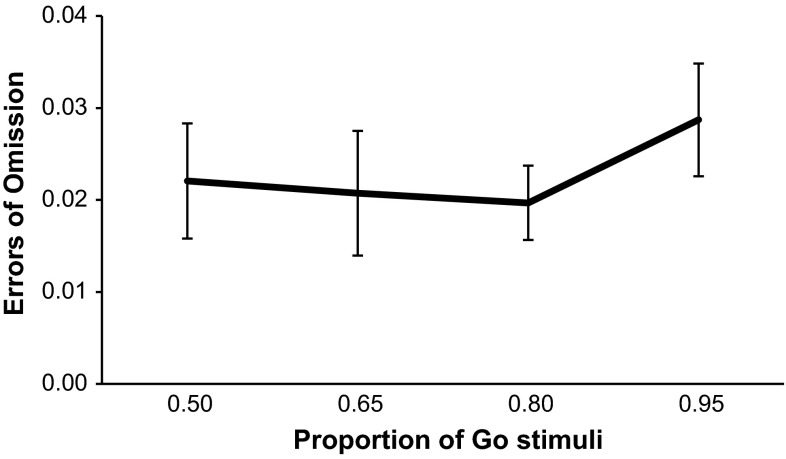
Mean proportion of errors of omission for each Go-stimuli proportion. *Error bars* are standard errors

For each subject in each Go probability condition, we calculated the proportion of commission errors (Fig. 1), the mean correct Go-stimuli reaction times (Fig. 2) and the proportion of omission errors (Fig. 3). One-way repeated-measures ANOVAs were performed separately on each of the three performance measures. The primary research focus was to test trends regarding the increase or decrease in the performance measures with increasing Go-stimuli probability (or decreasing No-Go-stimuli probability). We therefore used pre-planned orthogonal polynomial contrasts (Keppel and Zedeck 2001). These are 1-df contrasts in which concerns regarding sphericity assumptions do not apply. We limited our tests to the linear and quadratic trends, as we expected both linear and curvilinear trends. For errors of commission, there was a significant linear trend, *F*(1, 27) = 319.07, *p* < .001, $\eta_{p}^{2} = .922$, and a significant quadratic trend in the relationship, *F*(1, 27) = 10.45, *p* = .003, $\eta_{p}^{2} = .279$. As Go-stimuli proportion increased, so did errors of commission. For reaction times to the Go stimuli, there was a significant linear trend in the relationship, *F*(1, 27) = 236.47, *p* < .001, $\eta_{p}^{2} = .898$, and a significant quadratic trend too, *F*(1, 27) = 37.50, *p* < .001, $\eta_{p}^{2} = .581$. As Go-stimuli proportion increased, reaction times to the Go stimuli became faster. For errors of omission, there was no significant linear, *F*(1, 27) = .781, *p* = .384, $\eta_{p}^{2} = .028$, or quadratic trend, *F*(1, 27) = 1.99, *p* = .170, $\eta_{p}^{2} = .069$.

To further investigate the relationship between reaction time and errors of commission at each Go-stimuli proportion, a correlation analysis was performed with the mean commission errors and mean reaction times for each proportion (Table 1). At each proportion, the correlations are significant, *p* < .01. Furthermore, the association (*r*^2^) generally increases in strength as Go-stimuli proportion increases from .50 to .95.

**Table 1:** Correlation between reaction time and commission errors at each Go-stimuli proportion

Go-stimuli proportion	.50	.65	.80	.95
Correlation (*r* ^2^)	−.483	−.613	−.762	−.643

All *p* < .01

### Subjective state

**Fig. 4:**
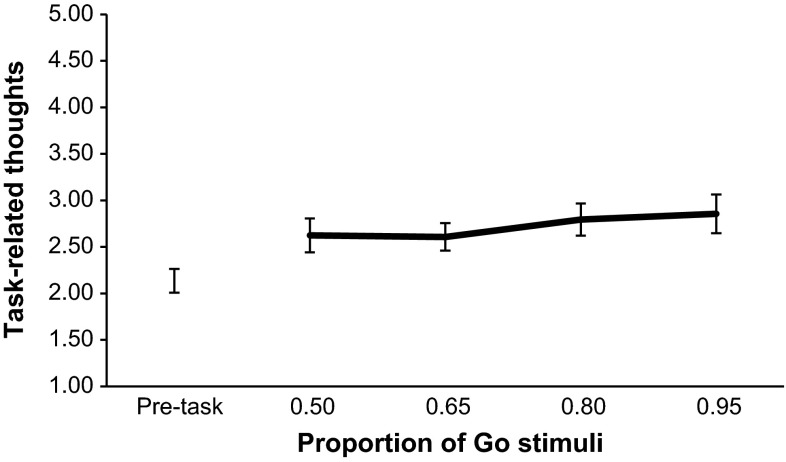
Mean task-related thoughts for each Go-stimuli proportion. *Error bars* are standard errors

**Fig. 5:**
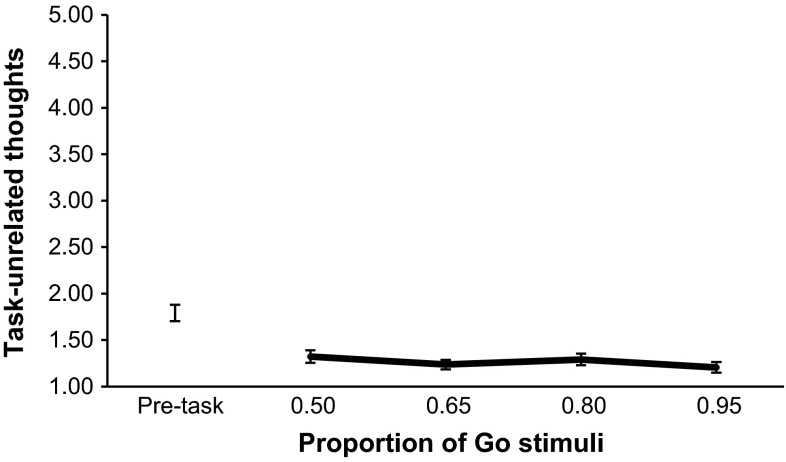
Mean task-unrelated thoughts for each Go-stimuli proportion. *Error bars* are standard errors

For each subject, we calculated the average scores on the two DSSQ subscales (task-related thoughts and task-unrelated thoughts), once before the tasks began (pre-task) and once after each of the four tasks, for a total of five measures. For both subscales (see Figs. 4, 5) we performed a one-way repeated-measures ANOVA. The assumptions of sphericity were checked using Mauchly’s test. This was primarily to test the pre-task questionnaire values with the post-task values. To test the differences amongst the different Go-stimuli probability conditions, we used pre-planned orthogonal polynomial contrasts as was the case with the SART performance metrics, excluding the pre-task baseline measure.

There was a significant effect of time on task-related thoughts, *F*(4, 108) = 6.32, *p* < .001, $\eta_{p}^{2} = .190$. Post hoc comparisons using the Bonferroni correction revealed that post-task task-related thoughts for both the .80 proportion and the .95 proportion were significantly higher than the pre-task task-related thoughts measure, *p* < .01. Polynomial contrasts with just the four different Go-stimuli probabilities revealed a significant linear trend, *F*(1, 27) = 5.03, *p* = .033, $\eta_{p}^{2} = .157$, with task-related thoughts increasing with increasing Go-stimuli probability. For task-unrelated thoughts, there was a significant effect of time, *F*(4, 108) = 25.93, *p* < .001, $\eta_{p}^{2} = .490$. Post hoc comparisons using the Bonferroni correction revealed that all of the four post-task task-unrelated thoughts measures were significantly lower than the pre-task task-unrelated thoughts measure, *p* < .01. Polynomial contrasts with just the four different Go-stimuli probabilities failed to show any significant trends. However, while not statistically significant, a potential linear relationship was observed in the direction of decreasing task-unrelated thoughts with increasing Go-stimuli probability, *F*(1, 27) = 2.88, *p* = .101, $\eta_{p}^{2} = .096$.

### Relationship between SART performance and subjective state

Both between-subjects and within-subjects correlations were investigated through the use of an established technique (see Head and Helton 2014; Zelenski and Larsen 2000). To investigate between-subjects correlations, each participant’s performance metric on the SART and their self-report responses (i.e. their mean average value for a condition) were averaged over the four conditions and then the correlations between these individual averages were calculated. Between-subjects correlations isolate the differences that can be attributed solely to trait individual differences after removing within-subjects variance. It is between-subjects correlations that are most commonly analysed in the SART (see Seli 2016). To investigate within-subjects correlations, for each condition each participant’s performance metric and self-report responses were converted to *standardized within*-*subjects z*-*scores* (see Head and Helton 2014; Zelenski and Larsen 2000) resulting in 4 reaction time *z*-scores, 4 commission error *z*-scores and so on for each of the metrics and questionnaire responses. The resulting *z*-scores were then combined across participants for the analysis or chained (see Head and Helton 2014; Helton et al. 2014). Table 2 displays the results of these analyses.

**Table 2:** Correlations between variables

	EC	EO	RT	TRT	TUT
Errors of commission (EC)		.144	−.920**	.296**	−.188
Errors of omission (EO)	.027		−.107	.152	.164
Reaction time (RT)	−.784**	.209		−.295**	.184
Task-related thoughts (TRT)	−.397*	−.437*	.229		−.092
Task-unrelated thoughts (TUT)	−.363	−.265	.170	.319	

Within subjects above main diagonal; between subjects below main diagonal

* *p* < .05; ** *p* < .01, for an *N* of 28

Errors of commission were significantly correlated with reaction time both within subjects and between subjects. At the within-subjects level, when a participant quickened their own rate of responding they were more likely to make a commission error themselves, and equally at the between-subjects level, participants who generally responded faster than other participants were more likely to also make more commission errors on average. Errors of commission were significantly positively correlated with task-related thoughts at the within-subjects level. When participants experienced an increase in thoughts about the task, this coincided with an increase in commission errors. At the between-subjects level, this result was reversed, with participants who reported higher task-related thoughts generally making fewer errors of commission. This was similarly seen with errors of omission, where participants who reported higher task-related thoughts also generally made less omission errors. Finally, at the within-subjects level increases in task-related thoughts within participants corresponded with them speeding up their reaction time to Go stimuli. Task-unrelated thoughts shared no significant relationships with any of the measures.

It was possible that the correlation between commission errors and task-related thoughts was influenced by participants’ response times, as has been noted before (Wilson et al. 2015a). To examine whether this was the case, a partial correlation was conducted. The partial correlation between task-related thoughts and errors of commission (between subjects) when controlling for response time was *r* = −.359, *p* = .066.

## Discussion

The current experiment investigated how performance on a high Go, low No-Go task and associated thought content changed as Go-stimuli proportion was manipulated across four conditions: .5, .65, .80 and .95 proportions. Measures of errors of commission, errors of omission and reaction times were taken to gauge SART performance, while two subscales of the DSSQ—task-related thoughts and task-unrelated thoughts—were used to measure self-reported thoughts.

The finding that as Go-stimuli proportions increased, reaction times decreased and commission errors increased could be accounted for by both the perceptual decoupling theory and the response strategy perspectives. The perceptual decoupling theory would suggest that the higher Go-stimuli proportions would have led to participants being lulled into more automatic responding, which should have resulted in either increased mindlessness or mind-wandering. If so, this should have been clearly reflected in the task-related thoughts and task-unrelated thoughts reported. However, this was not the case. Instead of task-related thoughts decreasing as Go-stimuli proportion became higher, they increased. Participants were evidently attentive to the task and therefore perceptually *engaged* rather than decoupled. Instead of task-unrelated thoughts increasing, as the perceptual decoupling view might predict, they appeared to actually decrease as Go-stimuli proportion increased. However, it should be noted that one interpretation of the perceptual decoupling model (mindlessness) might have successfully predicted the reduction in task-unrelated thoughts, but would have also predicted a decrease in task-related thoughts as well (true mindlessness—as in no conscious thoughts). Nonetheless, if participants had been perceptually decoupled from the task and engaged with mind-wandering, task-unrelated thoughts should have been positively associated with errors of commission and negatively associated with reaction time. Conversely, correlation analyses showed that task-unrelated thoughts shared no statistically significant relationship with commission errors or reaction times at either the within-subjects or the between-subjects level, and all four post-task measures of task-unrelated thoughts were significantly lower than the initial pre-task measure. Although not statistically significant, the direction of the correlations between task-unrelated thoughts and errors of commission and reaction times were actually in the opposite direction. Participants who reported more task-unrelated thoughts actually tended to make less commission errors (and this was true both within and between subjects). To the contrary, it was task-related thoughts that were significantly associated with errors of commission and reaction times at the within-subjects level and with errors of commission at the between-subjects level. This reflects the involvement or entanglement of the speed–accuracy trade-off with participants’ thoughts about the task, and this has also been seen in previous research with the SART (Wilson et al. 2015a). As to the cause of this relationship, it could be that participants who think more about the task then speed up their responding in an attempt to perform even better, but in doing so they inevitably have more difficulty withholding to the No-Go stimuli and thus make more commission errors. Or perhaps the act of making commission errors causes them to think more about the task (e.g. performance appraisal). Neither explanation fits with the perceptual decoupling idea. Instead, these explanations indicate participants’ conscious engagement with the task and are consistent with the idea that people are aware of the errors they make and that their reaction times over the task are contingent upon this awareness. Indeed, when a partial correlation was used to account for the influence of response time, the relationship between commission errors and task-related thoughts (between subjects) was no longer statistically significant (although the strength of the correlation was only slightly reduced). Regarding the task-related thoughts measure, it should be noted that task-related thoughts could in some circumstances be also classified as instances of mind-wandering. Some thoughts about the task may be due to a participant experiencing performance anxiety, for example worrying thoughts that their performance is poor. This highlights an area within the literature where further clarification is required. In the current study, we used task-*un*-related thoughts as the primary measure for off-task thoughts, as has typically been done (e.g. Birnie et al. 2015; Carter et al. 2013; Seli et al. 2015; Staub et al. 2014).

The differential effects that the varying Go-stimuli proportions had on commission errors and reaction time can be accounted for by the ACT-R model (Anderson and Lebiere 1998). The different Go-stimuli proportions each offered different opportunities in terms of the ideal response strategy for a given proportion. As expected, at higher Go-stimuli proportions participants favoured the encode and click strategy, evidenced by faster reaction times at the cost of more commission errors, whereas at lower Go-stimuli proportions participants used the encode and check strategy more often, demonstrated by slower reaction times but fewer commission errors (Peebles and Bothell 2004). Note that Peebles and Bothell’s (2004) account of SART responding bears similarities with the literature on other tasks requiring response inhibition, such as the stop-signal paradigm (see Logan 1994 for a review on this paradigm). Logan and Cowan (1984) found that increasing the probability that a participant would have to respond on trials (Go proportion) increased the strength of participants’ motor responses and impaired their ability to withhold the response when required. Ramautar et al. (2004) also observed that participants tended to sacrifice accuracy for speed when the probability of stop-signals (essentially No-Go trials) decreased. Much of the literature on response inhibition paradigms such as the stop-signal task may be concordant with research on the SART, particularly Peebles and Bothell’s account of SART responding. Indeed, it appears that response inhibition is firmly implicated in SART performance (Carter et al. 2013; Stevenson et al. 2011). It may be beneficial to attempt to consolidate these two different literatures in future.

The trend analyses provide a degree of support for the idea that a threshold exists wherein the feed-forward prepotent motor programme disproportionately increases in strength, leading to an abrupt shortening of response times and increase in commission errors. Commission errors had a curvilinear trend, whereby they initially gradually increased as Go-stimuli proportion became higher, but then increased markedly over the higher proportions, showing a similar pattern to that seen by Wilson et al. (2014). In their experiment they were unable to measure reaction time however, so the finding that in the present study reaction time exhibited a curvilinear trend and appeared to have an inverse relationship with errors of commission is notable. For the participant, a prepotent motor response ensures that the response is fast and without delay; however, it also makes withholding responses (when required) more difficult and therefore less likely. This is not the first time that an inverse relationship between speed and reaction time in the SART has been found. Head and Helton (2014) observed that SART performance oscillated over sessions spaced apart by a number of weeks. In sessions where participants tended to respond slower they made fewer commission errors, and conversely in sessions when they responded faster they made more commission errors. In the current experiment, the decreases in reaction time appeared to inversely mirror the increases in commission errors, with the biggest decrease between conditions appearing to occur between the .65 and .80 Go-stimuli proportions. This appeared to be reflected in the self-reported thoughts too. A visual inspection of the data for task-related thoughts (see Fig. 4 in “Results” section) suggested that the increase over Go-stimuli proportions was primarily due to a rise specifically between the .65 and .80 proportions. Although tests of this did not reach statistical significance, other tests did show that the only conditions where task-related thoughts were significantly higher than the pre-task baseline were the .80 and .95 proportions. In terms of the strength of the relationship between reaction time and commission errors at each Go-stimuli proportion, correlation analyses demonstrated that these associations generally became stronger as Go-stimuli proportion went from .50 to .95. In terms of the practical value of this in operational environments, being able to predict when a prepotent response may take precedence in an environment where a human is responding often and withholding responses less often, could be important information. For instance, a military or law enforcement commander may be able to use this knowledge to recognize when weapons operators are at a higher risk of committing friendly fire errors (see Wilson et al. 2013, 2014, 2015b). Nonetheless, it should be noted that tests for linear trends for reaction times and commission errors were also statistically significant in the current experiment, and further investigation of the disproportionate change in the SART performance metrics is required.

Correlation analyses revealed strong negative relationships, at both the within- and between-subjects level, between errors of commission and reaction time, the speed–accuracy trade-off. Errors of omission shared no association with reaction time and commission errors. There was, however, a significant negative correlation between task-related thoughts and omission errors and commission errors at the between-subjects level. People who tended to report more task-related thoughts tended to make less errors of omission and less errors of commission. Task-related thoughts had a significant positive correlation with errors of commission and a significant negative correlation with reaction time at the within-subjects level. When people tended to report more task-related thoughts, they tended to make more errors of commission and had faster reaction times. This is likely due to common fate with increasing Go-stimuli probabilities; increases in task-related thoughts were linked with increasing Go-stimuli proportions and probably occurred independently of the changes in the behavioural metrics. Participants tended with high Go-stimuli probabilities to respond faster, make more errors of commission and report more task-related thoughts.

Future research should look to further disentangle the nature of the notable decrease in people’s ability to withhold in Go/No-Go tasks, which appears to be closely related to the proportion of Go to No-Go stimuli and the response strategy used. In terms of the proportions that researchers should look to employ, using more intervals within the .5 to 1.0 Go-stimuli proportions should help to determine or narrow down the proportions of particular interest. A useful addition may also be a condition where 100 % of responses required are Go-stimuli responses. This will enable a ceiling reaction time to be established. Perhaps at a Go-stimuli proportion of .95, like the highest proportion that was employed here, participants are very close to a “ceiling” reaction time and physically at their limits anyway. However, the fact that participants in this .95 proportion condition were still able to occasionally withhold responses to No-Go stimuli, albeit only around 30 % of the time, suggests that their limits can be pushed further.

In the current experiment, participants appeared to adopt the response strategy that provided the most utility for each of the differing Go-stimuli proportions. In a high Go task such as the SART, response inhibition appears to be dictated by response strategy, and this in turn seems to be determined by, or at least strongly influenced by, Go-stimuli proportion. This relationship may become stronger when a virtual “breaking point” or threshold is reached. This threshold appears to be somewhere between the .65 and .80 Go-stimuli proportion. The functional relationship between Go-stimuli proportion and errors deserves further exploration. The findings of this research provide further evidence that performance on the SART, and perhaps by extension high Go/low No-Go tasks, is heavily influenced by response strategy and response inhibition. Sustained attention may nevertheless contribute to performance on the task; however, this may involve a form of internally directed attention (executive control is required to regulate response strategies) as opposed to externally directed attention (e.g. perceptual decoupling). Researchers who intend to measure externally directed attention, perceptual coupling, would be better served by using a different measure than the SART. The SART may be a useful tool for other purposes however, such as modelling behaviour in Shoot/Don’t-Shoot scenarios (Wilson et al. 2015b). Performance in the SART appears to be determined by a different measure (response strategy) than what many researchers are currently intending to measure (sustained attention). The findings here cannot easily be explained by a perceptual decoupling model. More specifically, the mind-wandering interpretation of SART performance errors requires closer scrutiny. The current findings are consistent with a theory of strategic responding in the SART. How consciously reported thoughts influence strategy choice in the SART remains a topic for future research.
